# On social psychiatry: Psychopathology of German justice on example of resident houses (RH)

**DOI:** 10.1192/j.eurpsy.2021.1879

**Published:** 2021-08-13

**Authors:** E. Neu, M. Michailov, J. Foltinova, C. Luetge, H. Schumitz, G. Weber

**Affiliations:** 1 Pharmaco-physiology, Inst. Umweltmedizin (IUM) c/o ICSD/IAS e.V., POB 340316, 80100 M. (Int.Council Sci.Develop./Int.Acad.Sci. Berlin-Innsbruck-Muenchen-NewDelhi-Paris-Sofia-Vienna), Muenchen, Germany; 2 Fac. Medicine, Univ. Bratislava, Bratislava, Slovak Republic; 3 Inst. Ethics, Techn. Univ., Muenchen, Germany; 4 Fac. Psychology, Univ. Luxemburg & Vienna, Vienna, Austria

**Keywords:** UNO-Agenda21, social psychiatry, justice psychopathology

## Abstract

**Introduction:**

Social PSYCHIATRY is essential for solutions of RH-conflicts leading to immense psychic&medical problems-[2a-e,3]. Millions tenants in Europe: Germany-54.3%/Austria-30.2%/France-25.3%/GB-24.1%/Italy-12.9%/Slovenia-4.5%. German journals reflect catastrophic situation of tenant-lessor conflicts-[3].

**Objectives:**

REFERENCES: [1a]-Luetge,Ch et-al(ed): Experimental Ethics, Basingstoke:Palgrave-Macmillan,2014. [1b]-Pegoraro,R (Chancellor Academy/Vatican-City) «Arzt und Christ» 38:3-55,1992; EACME-2017-Barcelona AB:p.129-130. [2]-Michailov,M.Ch, Neu,E, Welscher,U et-al: [2a]-Psychology: EFPA-2019-Moscow AB:p.1529,1530,1549. IUPsyS-2008-Berlin Int.J.Psychol. 43/3-4 p.154,248,615,799. [2b]-Psychiatry: EPA-2020-virtual/Madrid, Eur.Psychiatry 63S:EPP0834/5+EPV0581/1470; EPA-2019-Warsaw 56S:S689; EPA-2018-Nice 48/S1:S623&567&662. WPA-2019-Lisbon, E-Poster WCP19-2137/-1822/-1839: 2018-Mexico-City, Abs.-Book WCP18-0584/-0625/-0643/-0654. 2011-Buenos-Aires, AB:PO1.200. [2c]-Philosophy&Law: IVR-2019-Lucerne Progr.Book:p.114-116. FISP-2018-Peking Abstr.Book(AB)1348-50,1373-4,1420; -2013-Athens AB464-5/503-4/766. EACME-2017-Barcelona/MedEthics) AB73-74/125-126. [2d]-Psychosomatics: ICPM-2017-Peking AB:ID: 648493/648895/647749/648878; -2005-Kobe, JPsychosomRes 58: 85-86. [2e]-Physiology: DPG-2019 (German-Austrian-Suisse Soc.) Acta-Physiol., 227/S719, A03-3,A03-4,A03-9,A04-4,A05-1. IUPS-2017-Rio-de-Janeiro, AB:ID977; IUPS-2009-Kyoto, J.Physiol.Sci. Proc-IUPS-Vol.XXII/Springer,p.249. [3]-German-journals-“tz”-München, 14.02.2019, 15.02.17, 06.12.16/p.10, 18.10.16/p.10. Süddt.Zeitung-no172/p.30, 2017. Bild-14.12.2018/p.12. Mü.-Merkur-14./15.12.2019/no289/p.33.

**Methods:**

Psychological-medical-social observations-[2a,e].

**Results:**

Complex interaction of social-natural factors (micro-ecology/apartments) are demonstrated by conflicts tenants-lessors (RH-Munich). Conflicts conc. high-rents, luxurious repair, cause dangerous psychoneurological diseases: anxiety-neurosis-insomnia-depression,etc., esp. in patients/seniors with cardio-vascular pathology. Defect-doors&radiators&windows (air-currents) induce respiratory-diseases, defect-illumination causes accidents (neuro-orthopaedic diseases: commotio-cerebri,etc.). Examples for impossible situation in German-RH: After 47years annihilation of RH-contract (tenant-woman 74years); over 4.5years lessor tries to eliminate 2scientists from RH, living-working 40/50years (one invalid, other 86years, both with complex pathology) by justice-terror; RH-contracts of tenants 90years with dementia&blind-senior (90years) are annihilated. RH-conflict leads to lethal consequences of 73 year tenant-[3].

**Conclusions:**

SOCIAL PSYCHIATRY could help millions of tenants injured by RH-conflicts-[2a-c,1a,b] by (a)-psychotherapy&education considering “total symptoms of mind-body, acc. to Emperor AKIHITO during ICPM-2005-Kobe-[2d], (b)-education of RH-administrators incl. philosophical/psychological/psychiatric-examination, (c)-foundation of „house-councils“ for „RH-industry“ counteracting psychopathological/-somatic diseases. This way will be supported UNO-Agenda21 for better health/education/ecology on global-level.

**Disclosure:**

No significant relationships.

